# Primary Bovine Extra-Embryonic Cultured Cells: A New Resource for the Study of In Vivo Peri-Implanting Phenotypes and Mesoderm Formation

**DOI:** 10.1371/journal.pone.0127330

**Published:** 2015-06-12

**Authors:** Isabelle Hue, Danièle Evain-Brion, Thierry Fournier, Séverine A. Degrelle

**Affiliations:** 1 INRA, UMR1198 Biologie du Développement et Reproduction, Jouy-en-Josas, France; 2 INSERM, UMR-S1139, U767, Faculté des Sciences Pharmaceutiques et Biologiques, Paris, France; 3 Université Paris Descartes, Sorbonne Paris Cité, Paris, France; 4 PremUp Foundation, Paris, France; Faculty of Animal Sciences and Food Engineering, University of São Paulo, BRAZIL

## Abstract

In addition to nourishing the embryo, extra-embryonic tissues (EETs) contribute to early embryonic patterning, primitive hematopoiesis, and fetal health. These tissues are of major importance for human medicine, as well as for efforts to improve livestock efficiency, but they remain incompletely understood. In bovines, EETs are accessible easily, in large amounts, and prior to implantation. We took advantage of this system to describe, *in vitro* and *in vivo*, the cell types present in bovine EETs at Day 18 of development. Specifically, we characterized the gene expression patterns and phenotypes of bovine extra-embryonic ectoderm (or trophoblast; bTC), endoderm (bXEC), and mesoderm (bXMC) cells in culture and compared them to their respective *in vivo* micro-dissected cells. After a week of culture, certain characteristics (e.g., gene expression) of the *in vitro* cells were altered with respect to the *in vivo* cells, but we were able to identify “cores” of cell-type-specific (and substrate-independent) genes that were shared between *in vitro* and *in vivo* samples. In addition, many cellular phenotypes were cell-type-specific with regard to extracellular adhesion. We evaluated the ability of individual bXMCs to migrate and spread on micro-patterns, and observed that they easily adapted to diverse environments, similar to *in vivo* EE mesoderm cells, which encounter different EE epithelia to form chorion, yolk sac, and allantois. With these tissue interactions, different functions arose that were detected *in silico* and corroborated *in vivo* at D21–D25. Moreover, analysis of bXMCs allowed us to identify the EE cell ring surrounding the embryonic disc (ED) at D14-15 as mesoderm cells, which had been hypothesized but not shown prior to this study. We envision these data will serve as a major resource for the future in the analysis of peri-implanting phenotypes in response to the maternal metabolism and contribute to subsequent studies of placental/fetal development in eutherians.

## Introduction

Although differences exist among viviparous vertebrates (e.g., different fetal nutrition strategies, different placental origins and complexities), all are characterized by the close apposition and interaction (e.g., respiratory, nutritional) between maternal (uterine) and extra-embryonic structures during gestation. Moreover, among amniotes (reptiles, birds, mammals), extra-embryonic tissues (EETs) share the same ontogenetic origin and display the same four membranes (amnion, chorion, allantois, yolk sac [1]). In addition to supplying nutrients to the embryo, EETs contribute to early embryonic patterning [2], fetal tissue development [3], primitive hematopoiesis [4], *de novo* blood vessel formation–essential for chorio-allantoic placentas [5]–and to fetal health in response to maternal nutrition.

Within the EET, the chorion is a bilayer of ectoderm and mesoderm, while the yolk sac and allantois are bilayers of endoderm and mesoderm [6]. Among these extra-embryonic layers, the ectoderm (or trophoblast) is the most well-known, and has long been studied in mammals [7], while the endoderm has attracted more recent interest in the mouse due to its specification and differentiation patterns [8]. However, the extra-embryonic (EE) mesoderm, though essential to EET formation, has only rarely been studied at pre-placental stages [9, 10]. This may be due to the use of early implanting models in which it is not easily accessible (mouse, rat, human) or to its under-appreciated function in comparison to the embryonic mesoderm, which gives rise to a variety of cell types and organs [11].

Ungulates, however, are excellent models in which EE layers develop prior to implantation, are easily accessible [12], and available in large amounts (due to exponential growth, or elongation), so that all extra-embryonic cell types are accessible. In addition, in pigs [13], sheep [14], and horses [15], extra-embryonic mesoderm has been putatively observed prior to any sign of primitive streak formation, but not unambiguously shown, due to the absence of molecular markers for these early EE mesoderm cells [14]. In most other amniotes, mesoderm egresses the embryo from the primitive streak [16] as the result of a developmental Epithelial-to-Mesenchymal Transition (EMT) [17, 18] which gives rise to all extra-embryonic and embryonic mesoderm subtypes through a process that is spatially and temporally regulated [19] and controls the exit from pluripotency [11]. In view of the radial mode of mesoderm formation in turtles [20], its radial induction in chicks [20], or its radial differentiation from human embryonic stem cells grown on micropatterns [21], independently of primitive streak formation, it would be of great value to determine if the early EE cells that grow radially to the ED axis in bovines are mesoderm cells.

To begin deciphering extra-embryonic complexity prior to placenta formation, we isolated bovine extra-embryonic subtypes at Day 18 (D18), three days prior to implantation (D21), and characterized them using *in vivo*, *in vitro*, and *in silico* methods. Specifically, we were able to identify: i) “cores” of cell-type-specific and substrate-independent genes that were shared between *in vitro* and *in vivo* samples, ii) *in vitro* culture conditions that allowed bTCs to better resemble trophoblast cells from *in vivo* D18 EET, including both the mono- and bi- nucleated subtypes, and iii) new molecular markers to enable reconsideration of long-known features of *in vivo* EETs at D14-15 and D21-25 (see experimental design in S1 Fig).

## Results

### *In vivo* and *in vitro* extra-embryonic cell types

**Fig 1:**
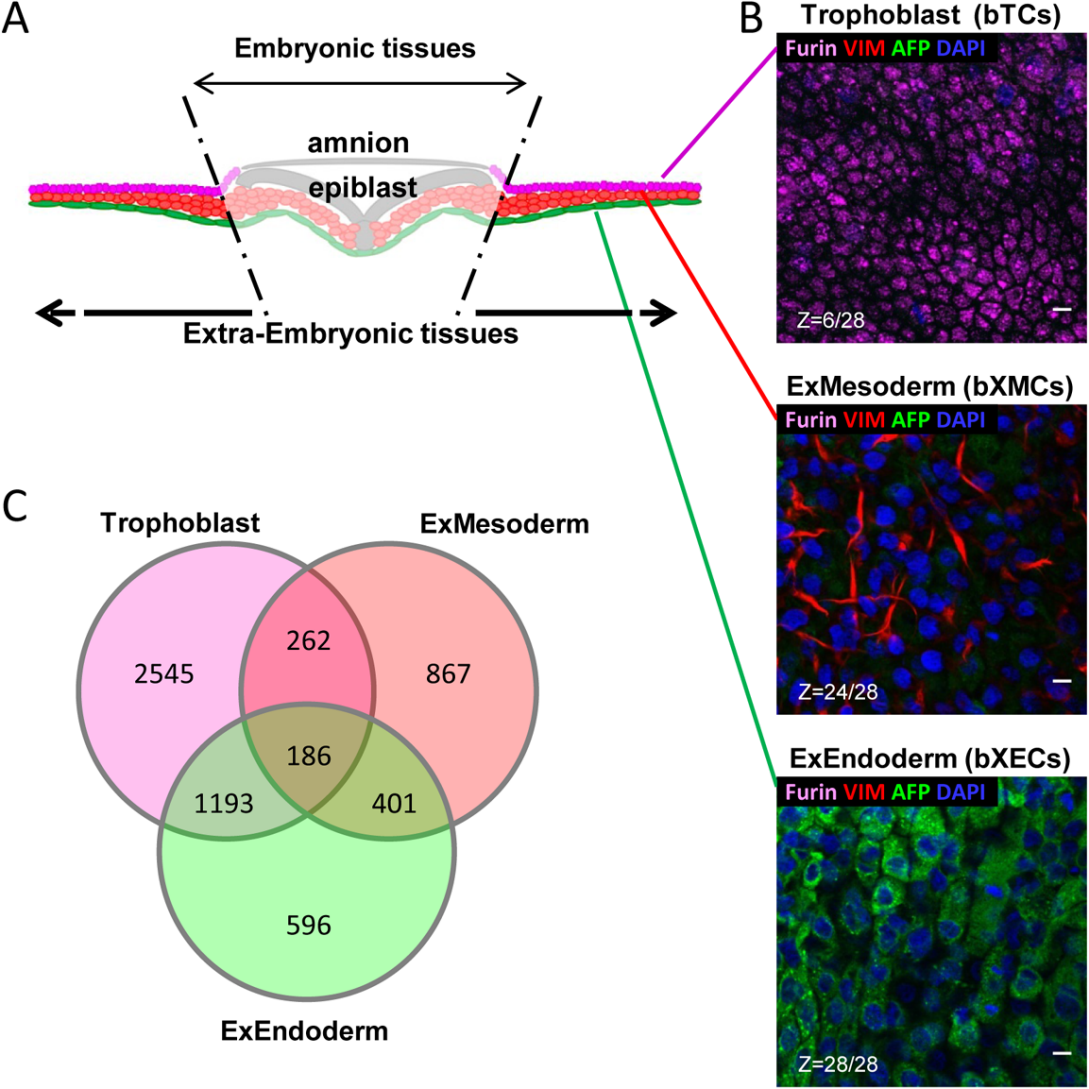
Phenotypes of *in vivo* micro-dissected cell types at D18. (A) Schematic view of the extra-embryonic tissues (EETs) that are in the vicinity of the embryonic tissues: ExEctoderm (or Trophoblast, in magenta), ExMesoderm (red), ExEndoderm (green). (B) Co-immuno-fluorescence and confocal microscopy (including z scans: Z) on whole D18 EETs with antibodies against reported *in vivo* markers for the Trophoblast (FURIN or PCSK3 [27]), ExMesoderm (VIMENTIN [66]), and ExEndoderm (AFP [67]). (C) Gene profiling using the bovine 10K array (GPL7417, [26]). All expressed genes were considered. Scale bar: 10 μm.

In a search for cell-type-specific phenotypes, we micro-dissected bovine extra-embryonic cells from the ectoderm/trophoblast (bTC), mesoderm (bXMC), and endoderm (bXEC) (Fig 1A), and assessed their features using validated markers (Fig 1B). *In situ*, trophoblast cells were small, containing intra-cytoplasmic dots of Furin; mesoderm cells had elongated forms and expressed Vimentin; while endoderm cells were larger in size, displaying large nuclei and cytoplasmic Alpha-Feto-Protein (AFP). A gene expression profiling analysis completed the initial characterization of each cell type and, as previously reported in the literature, trophoblast cells expressed *IFN-tau*, *PAG*s, and *PTG*s; mesoderm cells expressed *HAND1*, *IGF2*, and *MMP2*; and endoderm cells expressed *APLP2*, *DAB2*, and *FN1*. The main outcome of this analysis was that i) the trophoblast and mesoderm expressed high numbers of cell-specific genes, with the trophoblast expressing by far the most, ii) many genes were expressed in both the trophoblast and endoderm, and iii) the mesoderm shared more genes with the endoderm than with the trophoblast (Fig 1C, lists of all genes: Tables 1–7 in S1 File).

**Fig 2:**
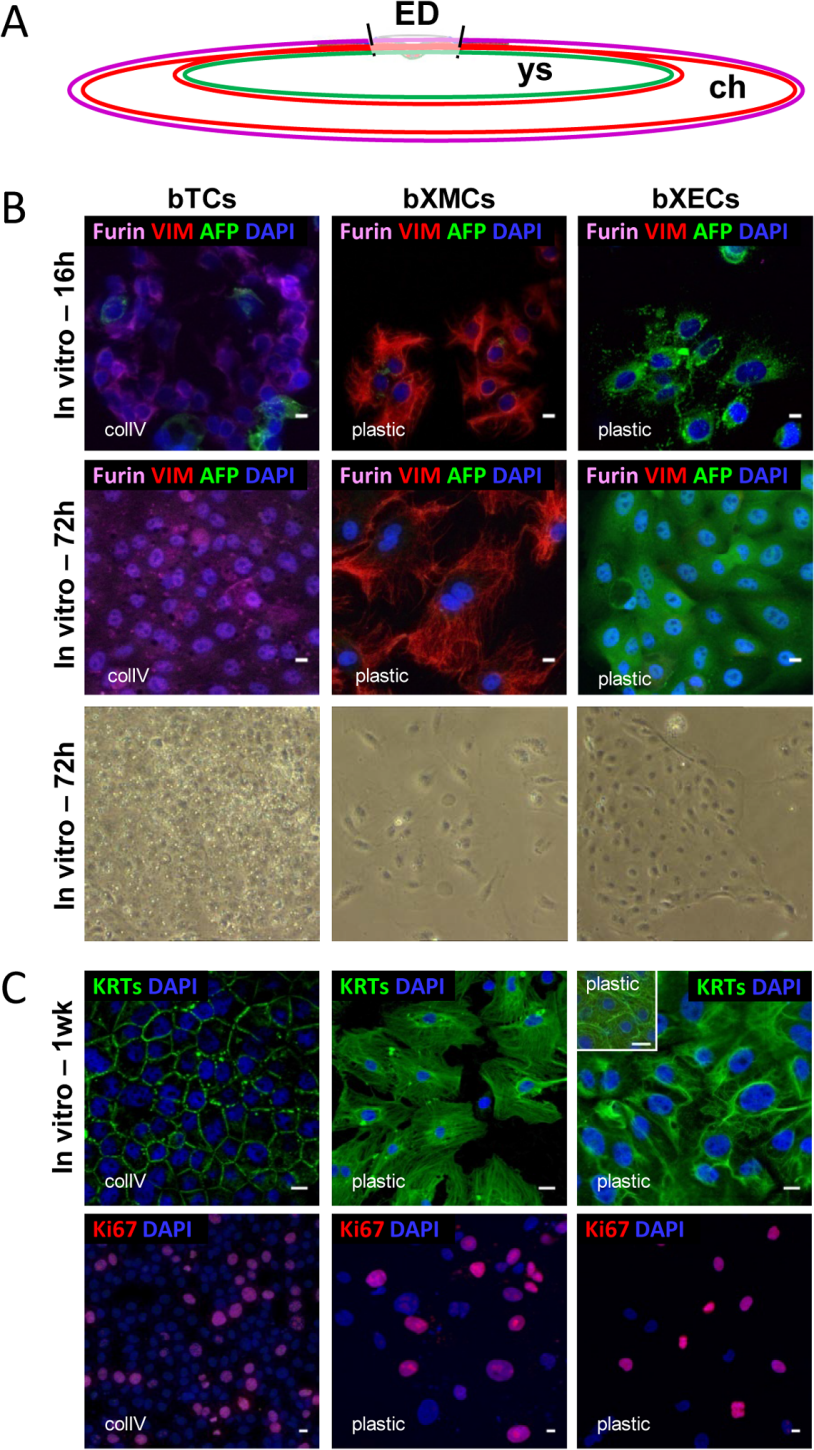
Phenotypes of *in vitro* cultured cell types at D18. (A) Schematic view of whole EET surrounding the embryonic disc (ED). Chorion (ch) is composed of ectoderm (or Trophoblast; magenta line) and ExMesoderm (red), yolk sac (ys) of ExMesoderm and ExEndoderm (green). (B) bTCs, bXMCs, and bXECs were primarily cultured on collagen (colIV) or plastic (where plastic means a tissue-culture-treated surface). Co-immunofluorescence is shown at 16h, 72h, or a week of culture with the antibodies used *in vivo* (Fig 1: Trophoblast—FURIN, ExMesoderm—VIM, or ExEndoderm—AFP) as well as phase-contrast images of each cell type after 72h of culture. (C) Immunodetection of cytoskeleton organization and cell proliferation with pan-Keratins (KRTs) and Ki67 respectively, after 1 week of culture. Scale bar: 10 μm.

To complement these molecular data with work at the cellular level, we used enzymatic digestions and density gradients to separate out the different cell types in Day 18 EETs (Fig 2A), then cultured the cells *in vitro*. Our characterization of these cells (Fig 2B) detected a minor degree of ectopic labeling in bTC or bXEC cultures at the beginning of the culture period (16h), but after 72h, each culture was enriched in only a single cell type. Within a week, cultures of each cell type displayed distinct cell shapes, cytofilament networks, and cycling cell ratios (Fig 2C). Notably, two phenotypes often occurred within bXEC cultures, a mono-nucleated phenotype and a multi-nucleated one (Figure A in S2 Fig); we observed a similar distinction *in vivo* between cells obtained close to the embryonic disc or far from it (proximal or distal; Figure B in S2 Fig). Because they were difficult to obtain on a regular basis, perhaps due to their size (Figure C in S2 Fig), multi-nucleated bXECs were not analyzed separately but grouped together with the other bXECs for the rest of the study.

### Different cellular basics *in vitro*

**Fig 3:**
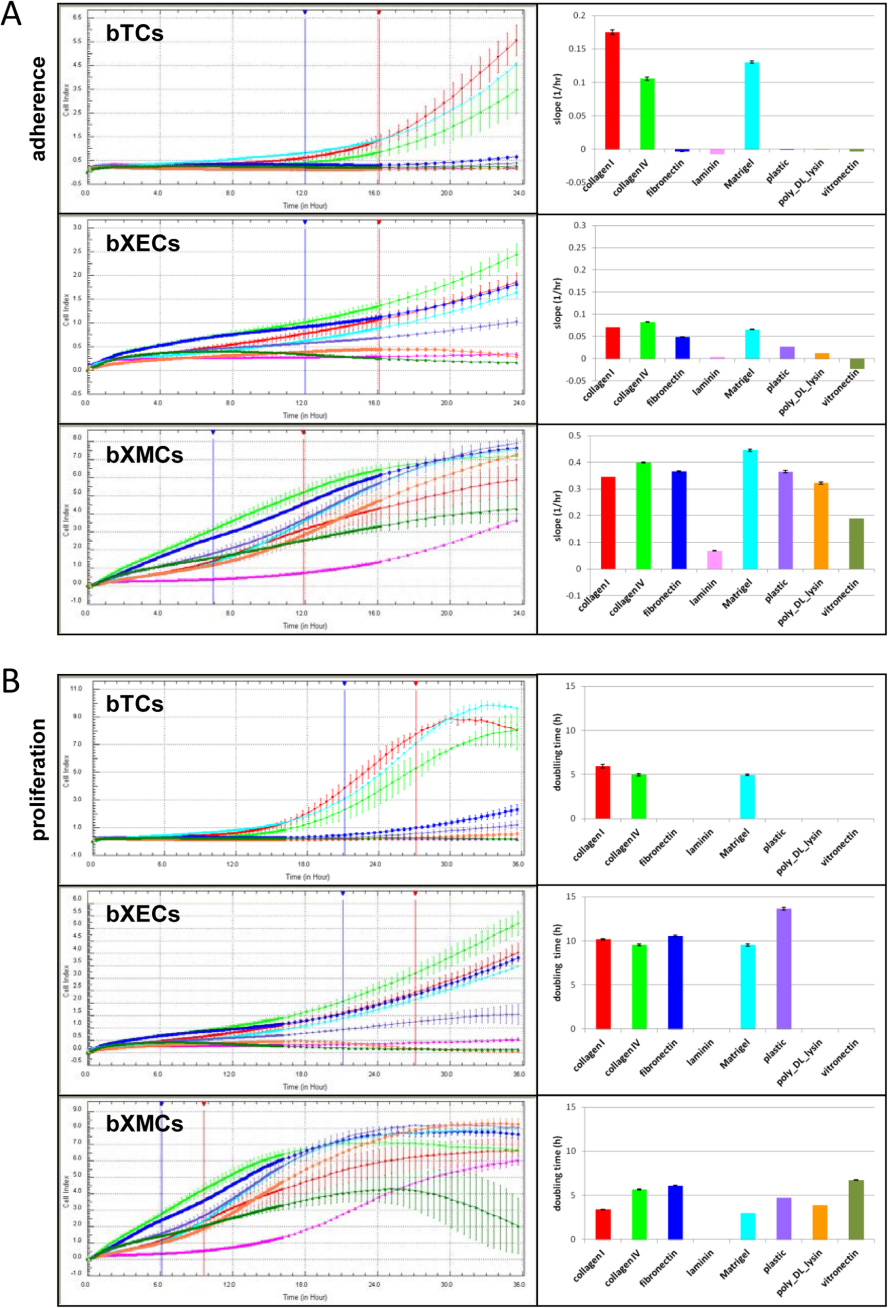
Dynamic monitoring for cell adhesion and proliferation. The adhesion and proliferation of bTCs, bXECs, and bXMCs on seven ECM components were individually monitored using the xCELLigence System; measurements were taken every 5 minutes during the first 16h and every 30 minutes during the last 24h of the assay. (A) Adhesion profiles are illustrated at the indicated time intervals (12–16 hours for bTCs and bXECs, 7–12 hours for bXMCs) on the left panels. For each cell type and ECM component, the adhesion index is indicated as the slope (1/hr; right panel). (B) Proliferation profiles are illustrated at the indicated time intervals (21–27 hours for bTCs and bXECs, 6–10 hours for bXMCs) on the left panels. For each cell type and ECM component, the proliferation index is indicated as the doubling time (right panel). The slope and doubling time of each growth curve were calculated using RTCA 1.2 software.

Next, we evaluated the adhesive and proliferative properties of each extra-embryonic cell type across a panel of extracellular matrix (ECM) components: collagen I, collagen IV, fibronectin, laminin, Matrigel, poly-DL-lysin, and vitronectin. As determined with impedance technology, each cell type had a specific adhesive profile and a specific adhesion kinetic for each ECM component (Fig 3A). For example, bTCs needed about 12h to spread on collagen I, collagen IV, or Matrigel, and had a 4-5h doubling time during their growth phase on these components (21-27h; Fig 3B). bXECs took about the same amount of time to seed into the wells but, unlike bTCs, also adhered to fibronectin and plastic. They grew slowly on these matrices, displaying a 10-12h doubling time during the early growth phase (21-27h; Fig 3B). In contrast, bXMCs easily adhered to all extracellular matrices (in less than 4h) with the exception of laminin, on which very little growth was observed in the first 16-18h. On all other matrices, bXMC doubling time during the early growth phase (6-10h; Fig 3B) varied from 3-6h depending on the ECM component: 3h on collagen I, Matrigel, and poly-L-lysin, but 6h on collagen IV, fibronectin, and vitronectin.

**Fig 4:**
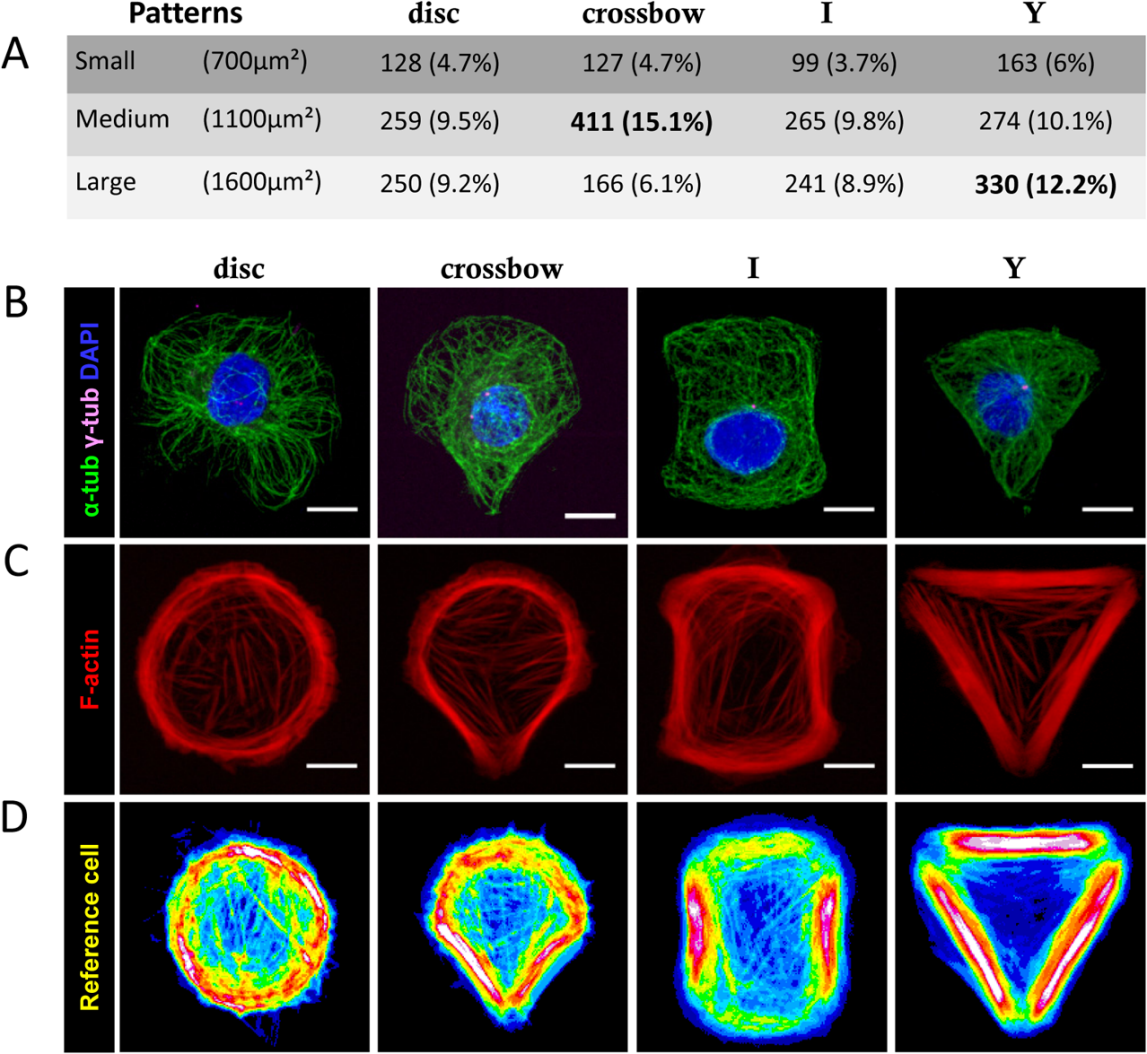
Plasticity of bXMCs. bXMC spread on four fibronectin patterns (disc, crossbow, I, or Y) of three sizes each [68]. (A) Number of cells that spread on each pattern. Cells were labeled with antibodies against (B) α and γ tubulins (green, magenta respectively), DAPI (blue), and (C) F-actin (red). (D) Over ten F-actin labeling images were averaged and color coded with the rainbow look-up table to highlight intensity (i.e tension) variations. Scale bar: 10 μm.

Because of its rapid adhesion to fibronectin (FN1; Fig 3A), FN1 micro-patterns were used to further evaluate the flexibility of individual bXMCs in four spatial distributions (a round shape, a crossbow shape, an [I], and a [Y]; on the same slide), each printed in three sizes. The best results, i.e. the highest percentage of cell spreading, was found with the medium-sized crossbow shape (15.1%) and the large-sized [Y] shape (12.2%), which both had an adhesion phase lasting only 1.5h. However, no pattern remained empty; at least 100 cells spread in each (i.e. 3.7%; Fig 4A). Depending on the spatial distribution of the FN1, we did see that cytofilaments, centrosomes, and nuclei organized themselves differently (Fig 4B), while stress fibers appeared stronger upon the non-adhesive edges of the cells (Fig 4C and 4D).

**Fig 5:**
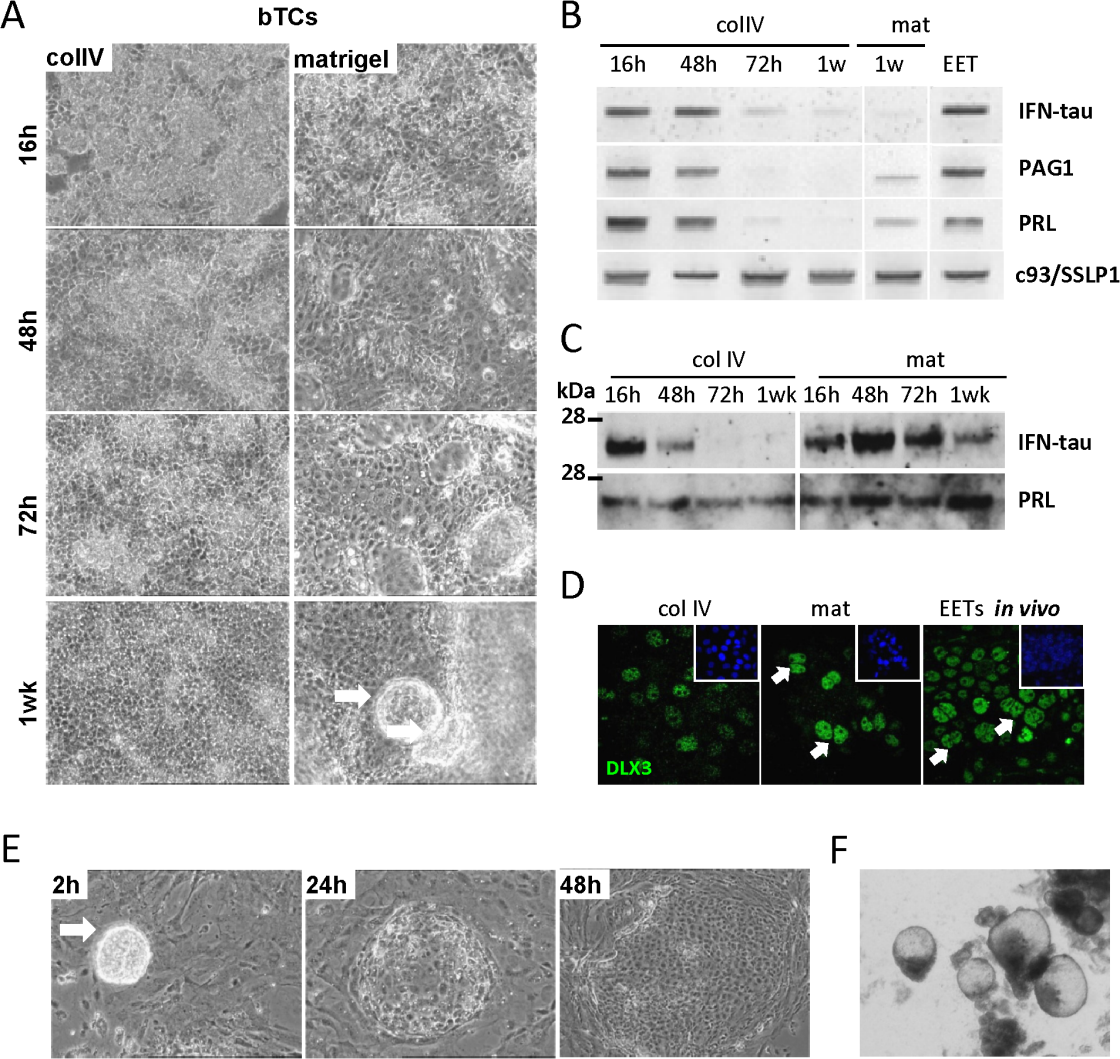
ECM composition affects gene expression in bTCs. (A) Phase-contrast images of bTCs after 16h, 48h, 72h, and a week of culture on collagen IV (colIV) or Matrigel (mat). Note the formation of a vesicle (white arrow) after a week of culture on Matrigel. (B) RT-PCR on *IFN-tau*, *PAG1*, *PRL*, and *c93/SOLD1/SSLP1* in bTCs cultured for 16h, 48h, 72h, and a week on collagen IV, as compared to week-old Matrigel cultures and to samples from *in vivo* D18 EETs. (C) Secretion of IFN-tau and PRL into the bTC culture media: 16h, 48h, 72h, and one week on collagen IV or Matrigel. (D) Immunofluorescent detection of DLX3 in bTCs after a week of culture on collagen IV or Matrigel, as compared to whole *in vivo* D18 EET. Note the strong expression of DLX3 in nuclei of the bi-nucleated cells (white arrows). (E) bTC vesicles (developed after 1 week on Matrigel) were co-cultured with bXEC. By 24h of co-culture, they had spread on top of the bXEC layer. (F) *In vivo* bTC vesicles, recovered from uterine flushes at D18.

Next, we further evaluated the *in vitro* response of bTCs to two different ECM components, collagen IV and Matrigel, and found substrate-specific modulation in gene expression, cell morphology, and cell fate (Fig 5). Specifically, we used reverse transcription (RT) PCR to examine four major genes of the bovine trophoblast: first, *IFN-tau*, which is expressed by the mono-nucleated cells (the major trophoblast subtype of D18 EET) and encodes a protein that is secreted at a high level a few days prior to implantation [22]; and also *Prolactin* (or *PRL* [23]), bo*PAG1* [24], and *DLX3* [25], which are specifically or highly expressed only in the bi-nucleated cells (the trophoblast cell type that appears by D18 *in vivo* and represents about 20% of this cell layer prior to implantation). Following 48h of growth on either collagen or Matrigel, we observed a decrease in *bIFNτ* expression (Fig 5B), but after a week of growth, a weak signal was still detectable. IFNτ was also secreted in the medium by bTCs grown on Matrigel for a week (23 kDa, as expected; Fig 5C), but cells grown on collagen IV decreased production of this protein by 48h, and it was no longer detectable at later measurement intervals (72h and 1wk). Similarly, *boPAG1* and *PRL* ceased to be expressed after 48-72h of growth on collagen IV, but were still detected on Matrigel after a week of growth (Fig 5B). Furthermore, PRL secretion (25 kDa) actually increased after a week on Matrigel (Fig 5C). Likewise, *Cited 1* (expressed in both cell types *in vivo* [26]) reached its peak expression after 1 week, which was even more than the expression levels in Day 18 EETs (not shown), whereas *c93* (*SOLD1/SSLP1*, specific to the mono-nucleated cell type [27]) was expressed at constant levels at all time points. Finally, DLX3 was expressed at higher levels in the bi-nucleated cells when bTCs were grown on Matrigel than on collagen IV (Fig 5D). Beyond this, though, bi-nucleated cells were neither counted nor separately analyzed, but incorporated into the rest of the bTC data. On Matrigel, bTCs also formed vesicles that detached from the monolayer and floated in the medium (Fig 5A and 5E), resembling those collected at low abundances in uterine flushes 18 days after artificial insemination (1.5%; Fig 5F). When co-cultured with bXECs, the vesicles spread on top of them in less than 24h (Fig 5E), mimicking what occurs *in vivo* at locations in the D18 EET where mesoderm cells have not migrated yet.

### Different gene cores *in vivo* and *in vitro*

**Fig 6:**
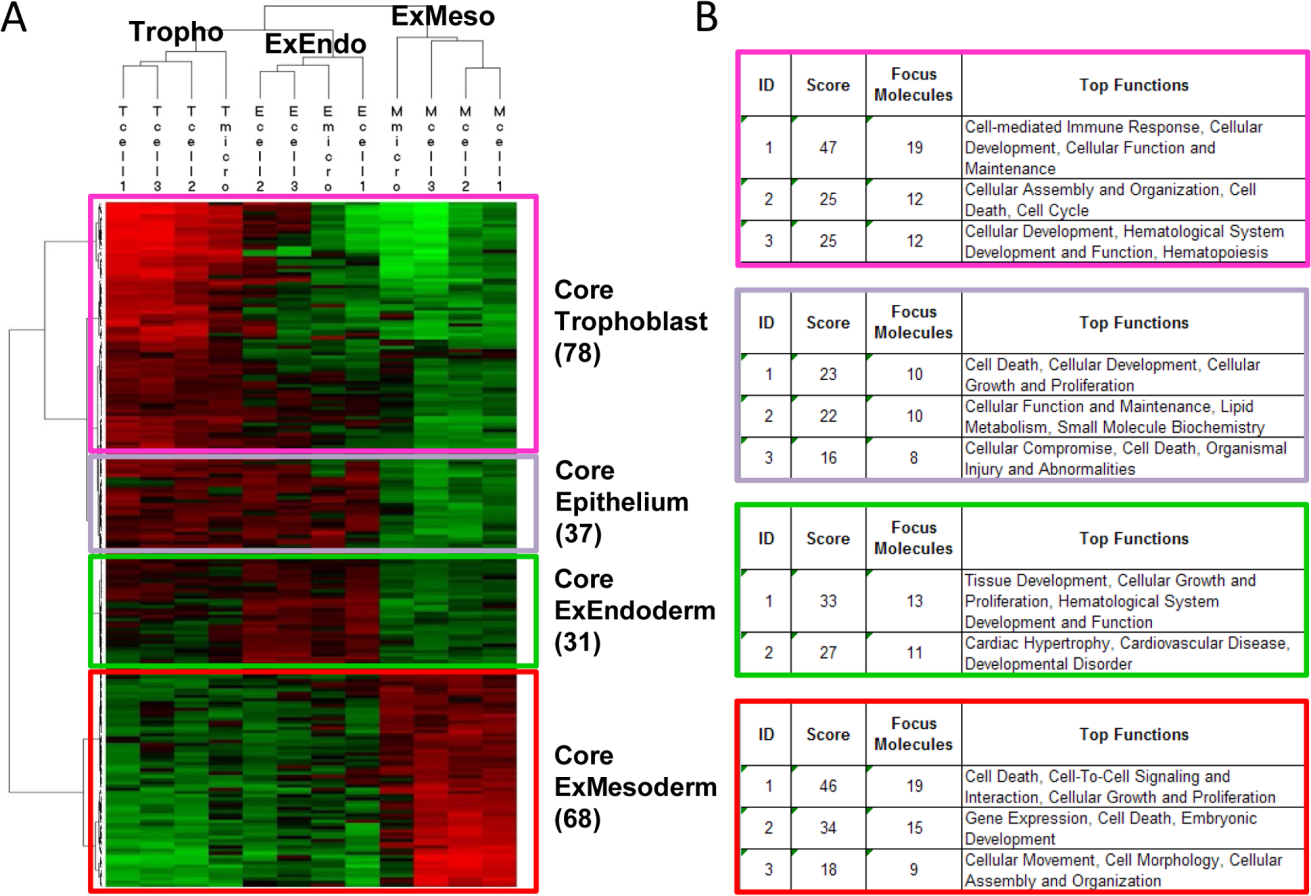
Differential gene expression among extra-embryonic cell types. (A) Hierarchical clustering and functions of 191 differentially expressed genes (DEGs). DEGs are clustered into three distinct cell types (Trophoblast, ExEndoderm, ExMesoderm) and four groups of genes (“core trophoblast”, “core epithelium”, “core ExEndoderm”, and “core ExMesoderm”). Samples are displayed in the vertical axis, genes on horizontal axis. Log2-transformed signal intensities are depicted with color: high expression levels in red, intermediate expression levels in black, and low expression levels in green. (B) Top biological functions of the four gene clusters using Ingenuity Pathway Analysis (IPA).

To learn more about each cell type and account for the confounding influences of ECMs, a gene profiling study was performed on bTC, bXEC and bXMC primary cell cultures and the *in vivo* micro-dissected cells from D18 extra-embryonic tissues. The results of this analysis showed that, for each cell type, the gene profiles of three independent cultures clustered together with their respective *in vivo* micro-dissected cell types (Fig 6A), regardless of the time point (3 days or a week) or the ECM substrate used (collagen IV, Matrigel, or plastic). In total, 191 differentially expressed genes (DEGs) were identified, which clustered into four main “cores”: trophoblast, endoderm, mesoderm, and epithelium (Fig 6A; Tables 1–4 in S2 File). A few DEGs did not cluster into one of the cores, suggesting that these genes were expressed in multiple cell types. However, the number of DEGs shared among cell types here was much lower than the number of shared genes in our previous gene expression analysis of *in vivo* micro-dissected cells (Fig 1C, Tables 1–7 in S1 File), because that initial analysis included all expressed genes, while our second analysis was restricted to only those genes that were differentially expressed.

The genes in the “Trophoblast Core” included many that have been reported in elongating trophoblasts (D12-D18: *AKR1B1*, *GATA2*, *GATA3*, *PAG2*, *PCSK3*, *TKDP* [28]), trophoblast cell lines (bovine CT1, D10-11: *GATA2*, *GATA3*, *PAG* [29]), early trophectoderm (bovine D7 blastocysts: *FABP5*, *GATA2*, *SLC15A1* [30]), or chorionic trophoblast stem cells (*PEG3* [31]). Most genes, however (41 out of 56), were new in the bovine trophoblast, including some involved in cell adhesion regulation and epithelial sheet morphogenesis. Genes in the “Endoderm Core” were mostly unreported from the EE endoderm, with 9 out of 24 linked to the regulation of cell proliferation and cytokinesis. Altogether, bovine EE endoderm cells shared more genes with mouse visceral subtypes (VE and ExVE: *APOE*, *EHF*, *ELF1*, *FN1*, *GATA6*, *IGF2*, *IRF2BP2*, *PDGFRA*, *PLAU*, *SDC4*, *TCF19*, *VEGFA*) than with the mouse parietal subtype (*LAMB1*, *SPARC*, *STRA6*) or mouse endoderm stem cells (*DAB2*, *NOTCH2* [32]). The genes from the “Epithelium Core” were expressed in both endoderm and trophoblast cells, including genes for ECM adhesion (*ITGB2*), cell junctions (*CRKL*, *GJB5*, *RABAC1*), epithelial functions (*BTBD7*, *MYO9B*), metabolic processes, or nutrient transport. The “Mesoderm Core” also contained genes previously unreported in the bovine EE mesoderm, involved either in histone modification or in the cell response to mechanical stimulus (n = 12/48). Altogether, bovine mesoderm cells shared genes with *in vitro* differentiated mesoderm (from mouse embryonic stem cells; *CDX2*, *MEST*, *TBX3*, *WNT5B*), mesendoderm (*PDGFRA*), human *in vitro* mesoderm progenitors (*CD56/NCAM1*, *HAND1*, *TGFBR1*, *VIM*), and bovine *in vivo* extra-embryonic mesoderm (*HAND1)* [9–11, 27, 33].

### Unique mesoderm signatures

**Fig 7:**
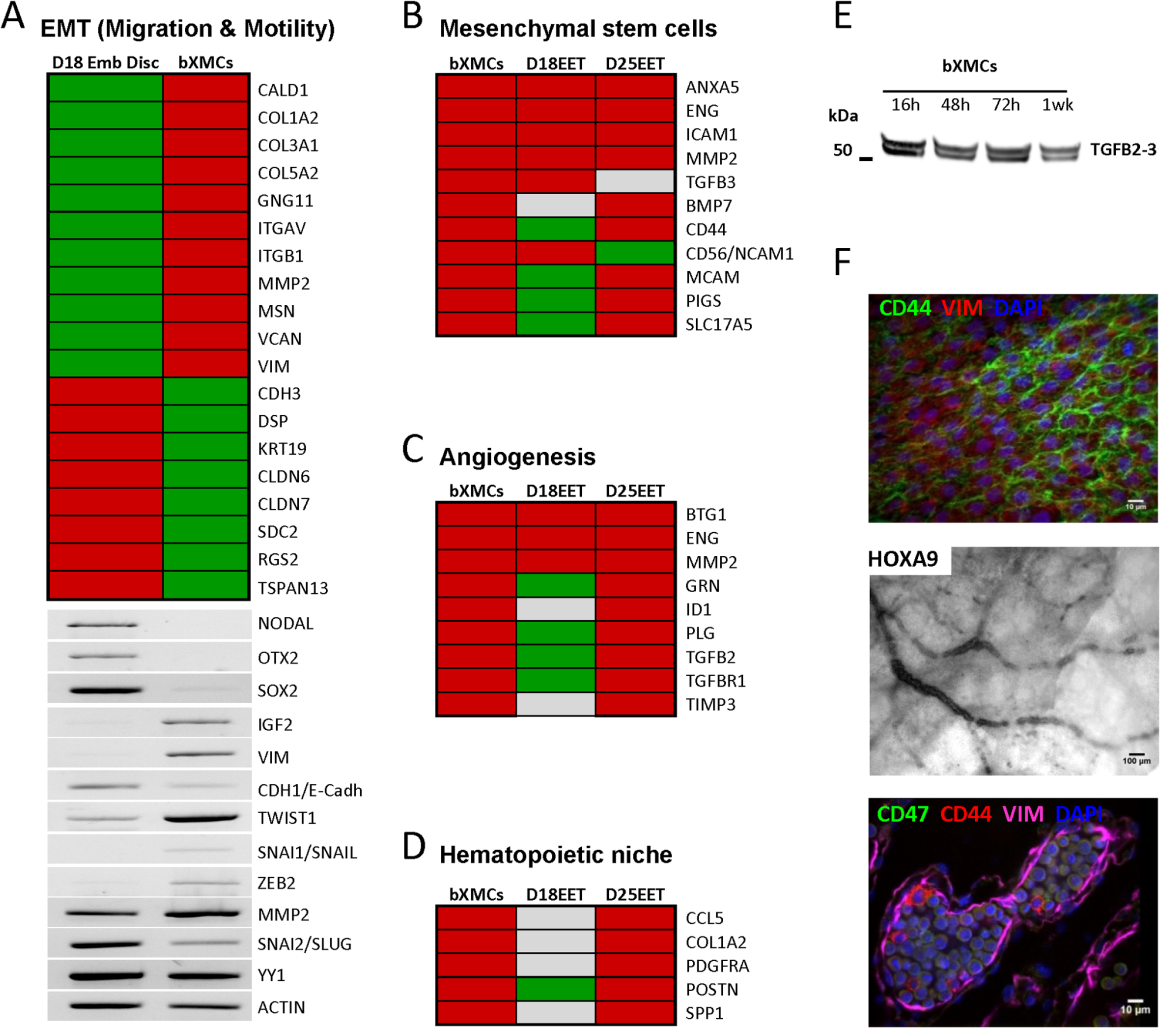
Unique features of D18 bXMCs. (A) Microarray analysis (upper panel) and RT-PCR (lower panel) demonstrate gene expression changes typical of a developmental EMT (from an embryonic disc at D18 to XMCs). (B-D) Microarray data reveal signatures associated with mesenchymal stem cells, angiogenesis, and hematopoietic niche in bXMCs and *in vivo* D18-D25 EETs (GSE13013). (E) Western Blot analysis shows TGFB2/3 precursor secretions by bXMCs cultured for 16h, 48h, 72h, and a week on plastic. (F) Consistent with *in silico* data, we found evidence of blood vessels in the allantois (as revealed by the expression of the *HOXA9* pro-angiogenic transcription factor [69, 70]) and blood islands in the yolk sac (as evidenced by a vitelline and a mesenchymal layer (VIM^+^) that surrounds erythrocyte precursors, i.e. round nucleated erythroblasts expressing more CD47 than CD44 [71–73]).

Beyond the above-mentioned differences, the “Mesoderm Core” was the only cluster for which one of the top-listed functions was “cellular movement”, a logical result given the fact that XMCs in amniotes originate from an EMT process. When compared to bovine embryonic discs, bXMCs showed increased expression of key EMT regulators (*SNAI1*, *TWIST1*, *ZEB2*) and effectors (e.g., *VIM*, *MMP2*; Fig 7A), together with decreased expression of *CDH1*, *TSPAN13*, or *VCAN*, and genes associated with cytoskeleton reorganization (*KRT19*, *MSN*, *VIM)* that altogether allow epithelial cell remodeling, delamination, and migration. Consistent with this observation, bXMCs migrated slowly on FN1 tracks (movie from the World Cell Race 2011, using a time lapse recording system; S3 Fig), but divided on Matrigel.

The Mesoderm Core also contained DEGs linked to mesenchymal stem cells (n = 10; e.g., *CD44*, *TGFB3*), angiogenic processes (n = 9), or the hematopoietic niche (n = 5; *CCL5*, *COL1A2*, *PDGFRA*, *POSTN*, *SPP1*). These genes also appeared in *in vivo*-developed EETs at D18, D25, or both, as confirmed with a dataset from a previous study (GEO accession number GSE13013; Fig 7B–7D). These *in silico* data were further supported ith biological data from both *in vitro* and *in vivo* samples demonstrating that: i) TGFB2/3 precursors were secreted by bXMC cultures, ii) CD44 was expressed in the chorion, iii) the formation of blood vessels and blood islands was observed in the yolk sac (Fig 7E and 7F), and iv) *COL1A2* has been previously reported in EET [34].

**Fig 8:**
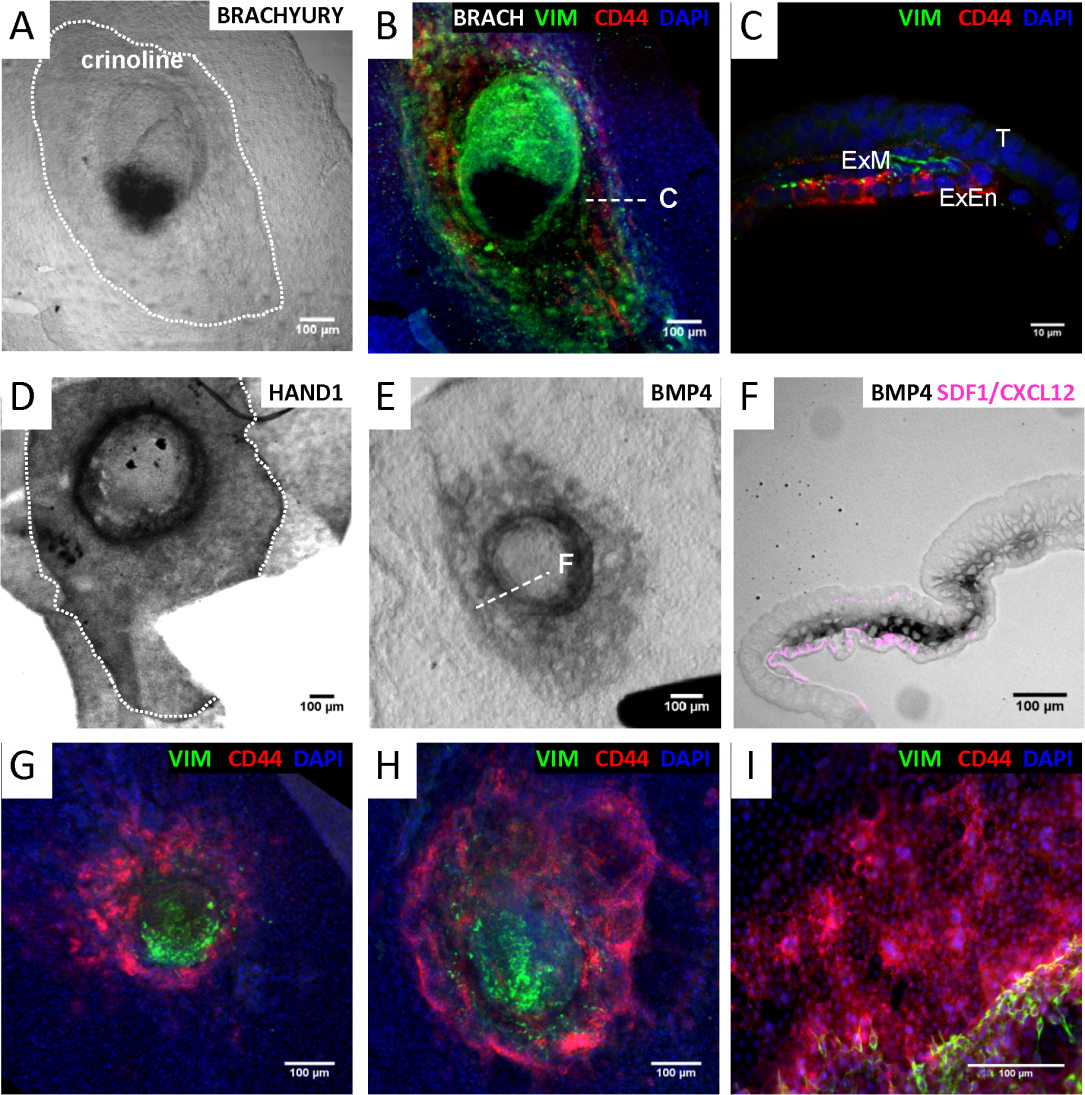
Nascent mesoderm and crinoline formation. (A, D, E) Whole mount *in situ* hybridization (WISH) with Brachyury, HAND1, or BMP4 DIG-labeled riboprobes. (B) Brachyury WISH with VIM, CD44, DAPI co-immunofluorescence. (C, F) Cross-sections from tissues in B and E, respectively. (F) SDF1 (CXCL12) immunostaining after a BMP4 WISH. (G-I) VIM, CD44, DAPI co-immunofluorescence. A to F: Dorsal views, G to I: ventral views. T: trophoblast, ExEn: extra-embryonic endoderm, ExM: extra-embryonic mesoderm.

Given the extent to which bXMC cultures shared patterns of gene expressions with D25 EETs, we searched for genes that might be shared between bXMCs, bXECs, and earlier-developing EETs, specifically: i) those at D15, when EE mesoderm cells are forming, originating by EMT from the epiblast of the embryonic disc (ED), and ii) those at D12, prior to EE mesoderm formation. To do so, we qualitatively assessed the gene expression profiles of EETs and EDs at D12 and D15 (data not shown), compared them to the profile of the *in vitro* cultures, and selected genes that seemed most relevant for a basic comparison of D12 to D15 EDs. As a result, the genes that appear to play an important role in early EE mesoderm formation are *CD44*, *HAND1*, and *SDF1*: *HAND1* was expressed by bXMCs and *in vivo* D15 tissues (ED+EET), while *CD44* and *SDF1* were expressed by bXECs and *in vivo* D12 tissues (ED+EET). Using these three genes and two mesoderm markers (*Brachyury*, *BMP4*), we analyzed the bovine early EE mesoderm in order to compare our results with those previously obtained from sheep, in which early EE mesoderm was described as i) appearing at a pre-streak stage and ii) being mesoderm due to its histological position, though negative for two early mesoderm T-box genes (*Brachyury* ^*−*^ *Eomesodermine* ^*–*^) [14]. We first observed at D15 that the EE mesoderm–surrounding the ED on its ventral side and extending in the elongation axis like the crinoline of dresses from the 19th century–did not express Brachyury transcripts but did express HAND1 mRNA (and VIM), while the EE endoderm expressed CD44 (Fig 8A–8D). At an earlier D15 stage, EE mesoderm cells expressed BMP4 transcripts, though no “crinoline” and no streak were morphologically detectable yet (Fig 8E). At both stages (crinoline: +/-), we noticed that the EE endoderm expressed: i) SDF1 (Fig 8F) and ii) CD44 prior to, and concomitantly with, EE mesoderm migration (VIM^+^ cells; Fig 8G–8I).

## Discussion

We isolated, cultured, and characterized the cell types that make up bovine EET three days prior to implantation. We used a complementary approach which utilized both dynamic monitoring of cellular properties as well as gene profiling data, and our results make a substantial contribution to knowledge of these cell types, particularly through the gene “cores” that identified (*in vivo* as well as *in vitro*) previously undescribed genes in the trophoblast, mesoderm, and endoderm that were related to cytokinesis, cell response to mechanical stimuli, epithelial functions, and nutrient transport. Additionally, the cultured cells developed for this study serve not only to supplement the panel of pre-existing trophoblast cells, but also to address the paucity of EE endoderm cells and, more importantly, the lack of EE mesoderm cells. These cell lines, together with the markers used here, provide new tools to revisit long-known histological features of D15 to D25 EETs.

### Unique cell types three days prior to implantation

Bovine trophoblast cell lines (mostly long-term cultures) have been previously described at both pre- and post-implanting stages [35], but such data are scarce for endoderm cells (characterized at earlier stages only: D7-8) and lacking entirely for mesoderm cells. In addition, despite sharing the “pre-implanting” descriptor, cells derived from these stages can vary significantly, as developmental differences are considerable between D7 and D18: D7-D10 embryos are spherical blastocysts while in D12-D18, they are already highly differentiated [36], and can in fact be 5–15 days post-implantation in rodents, primates, or lagomorphs. Therefore, cell derivation at these stages does not lead to equivalent phenotypes, as confirmed by a comparison of trophoblast CT-1 cells (derived from D10-D11 embryos [37]) with ours. The former resembled those of an ovoid stage (D12-D13 [36]), while the latter resembled those of a filamentous stage (D18) (bi-nucleated cells; *DLX3*, *PAG1*, *PRL* expression). Similarly, some of the bXECs displayed a multi-nucleated phenotype (S2 Fig) that had not previously been observed in endoderm cell cultures (D10-12; cow or pig endoderm: CE-1, CE-2 [37]; PE-1, PE2 [38]). Thus, the bTCs and bXECs developed here reveal their value in the novel phenotypes exhibited (molecular and cellular) and the fact that these cell lines were obtained from the same EET and from few culture passages (when others often exceed 50). In the future, these cell lines will be indispensable in efforts to decipher, both *in vivo* and *in vitro*, the complexity of peri-implanting extra-embryonic tissues.

### Confusing markers or partial knowledge?

In this work as in others, some reputed *in vivo* markers, such as *IFN-tau* (Fig 5), disappeared *in vitro* after a few days. Similarly, several observations, from the literature and from the present study, call into question current techniques/markers for identifying trophoblast cells. First, trophoblast cells that were derived from early embryos (D7-8) and cultured over 50 passages [35] expressed late pre-implanting markers that the original biopsies did not. Second, bTCs grown on Matrigel closely resembled *in vivo* EET (D18) due to the growth factors/chemokines [39] influencing bTC responses (Figs 4 and 5). Finally, CT-1 trophoblast cells (D10-11) that were co-cultured with endometrial cells or incubated with uterine flushes mimicked cells in the implantation stages (D22-23, [40]). With regard to the debate about defining trophoblast identity, both *in vivo* and *in vitro* [41], we thus pose the question: are a few markers enough, is a larger network needed, or is an epigenetic signature better? Obviously, in the current work, some markers (in the trophoblast core) were maintained *in vitro* better than *IFN-tau* was, which prompts us to suggest a new approach for cell-type definition: first, to define robust sets of markers for each EE cell type, as proposed here with the *in-vivo*/*in-vitro*-conserved “cores”, and second, to decipher gene regulation networks (transcriptional, epigenetic [41]) in order to improve knowledge of basic EE cellular behaviors in diverse micro-/macro-environments.

Beside cell-type-specific patterns, we also observed ones in common: shared by all cell types, by endoderm and mesoderm, or by ectoderm and endoderm (epithelium core; Fig 6). For example, primitive endoderm and nascent mesoderm both originate from the embryonic lineage, at D8-9 and D14-15, respectively. In sharing a common origin and migratory phenotype, they also share markers (e.g., *TIAM1*, *ZYX*). However, endoderm cells retain their epithelial nature and harbor an intermediate (or “squamous”) phenotype [42]. An intermediate phenotype is also present in implanting trophoblast cells due to a partial EMT (D22) but at D17 [43], EMT markers identify only mesoderm, rather than trophoblast, cells (Fig 7). Long-known from histological studies but poorly described at the molecular level, this shared squamous pattern may have its origins in the epithelium core described here of genes shared between ectoderm and endoderm. This reinforces the need for studies that clarify the landscape of EE markers across elongation and implantation stages.

### Cell-type-specific cellular biologies

The improved performance of bTCs on collagen substrates derives from the activity of ITGB5 (a reputed pan-trophoblast adhesion receptor [43, 44]) but also of numerous components [45] of integrin-mediated adhesion (adhesome) or focal adhesion (FA), among which: actin regulators (*LASP1*), adaptors (*GN2BL1*, *GRB2*, *GRB7*), GAPs (*DLC1*, *GIT1*, *RASA1*), GEFs (*ARHGEF12*), or Tyr-phosphatases (*PTPRF*, *PTPN6*). Consistent with their slow rate of spread and columnar nature *in vivo*, bTCs have been observed to express the highest number of cadherin-adhesion genes (cadhesome: e.g., *DLC1*, *GJB5*, *SHROOM3*, *TJP2*) and form vesicular structures when ECM-cell contacts weaken [46].

In contrast, bXMCs easily spread and proliferated over several substrates, including fibronectin. This is likely because they express integrins (*ITGAV*, *ITGB1*) that cooperate during rigidity sensing [47] and the LIM^+^ genes that mediate mechanotransduction (e.g., *TES*, *ZYX* [48]). Indeed, *in vivo* EE mesoderm cells sense their environment and react to it, changing their geometries in space or time thanks to their acto-myosin cytoskeleton or to the MMP proteolysis of the ECM components [49]. They can thus appear either rounded or elongated (VIM^+^ cells; Fig 8H and 8I). In addition, bXMCs expressed SRF-target genes (*VCL*, *WDR1* [50]) and migrated individually (S3 Fig) like embryonic mesoderm cells [50], or in chains (Fig 8) like mesenchymal cells [51].

*In vitro* monitoring of cellular dynamics can mirror the *in vivo* interplays and inputs from cell-ECM and cell-cell contacts [52]. This was demonstrated here by the performance of each extra-embryonic cell type on laminin (Fig 3), an ECM component involved in the regulation of EMT [53] on which only bXMCs were able to grow. Such monitoring efforts, particularly when linked with gene profiling, have the potential to reveal much about the fine-tuned processes that govern EET development.

### bXMC applications for the study of *in vivo* EE development

Because D18 bXMCs shared patterns of gene expression with D15 and D25 *in vivo* EETs, we used these cultures to facilitate an examination of primitive hematopoiesis/angiogenesis and EE mesoderm formation.

Consistent with the mesoderm’s contribution to early post-implantation EET functions [54], we identified molecular signs of a hematopoietic niche, blood islands, and early blood cells in the bovine yolk sac (Fig 7F). In addition, the expression of *HOXA9*, which is considered pro-angiogenic, in the proximal allantois coincided with an angiogenic process. *HOXA9* is unusual in that it is physically located with other *HOXA* genes at the 5’ end of the *HOX* cluster [55] but unlike its neighboring genes, had not been previously reported in murine or bovine allantoises. Another gene, *CD44*, was expressed at D25 in chorionic cells of *in vivo* bovine EETs; this gene is a reputed marker for mesenchymal stem cells, endothelial cells, and angiogenic-promoting activities [56]. As indicated by the above outcomes, researchers who attempt to isolate early post-implantation mesoderm subtypes will benefit from the choice of appropriate core genes (for example, CD56; S4 Fig).

Concerning EE mesoderm formation, we showed that: i) CD44 expression accompanied or preceded VIM^+^ cell migration towards extra-embryonic territories and ii) EE mesoderm cells were in contact with SDF1^+^ EE endoderm cells (Fig 8). Even though CD44 cleavage by MMP9 was reported to be favorable to cell migration, necessary to connect ECM and intra-cellular signaling [57], and essential to allow the fine-tuning of this signaling by ECM/BM (basement membrane) components in mouse embryos [58], this is the first time a study has shown CD44 and SDF1 expression at this stage in bovine embryos. Notably, SDF1 and its receptors (CXCR4, CXCR7) have been reported to be cues for directional migration in other tissues or species [59, 60], so finding them at the endoderm/mesoderm interface in bovine EETs is very promising. Nonetheless, in the absence of time-lapse imaging or functional studies, one may only hypothesize that MMP2-mediated proteolysis of SDF1 (CXCL12) favors EE mesoderm migration, or that constitutive SDF1 expression is transformed by the migrating cells (*CXCR7* ^+^) into a directional cue by SDF1 receptors (CXCR4, CXCR7 [60]). In support of this hypothesis, though, bovine EE mesoderm cells in the present study expressed both MMP2 and CXCR7. SDF1 and CD44 expression by EE endoderm could thus help the migration and/or differentiation of EE mesoderm cells.

As shown here, the EE crinoline or ring that surrounds the ED (before a streak is morphologically visible) is composed of cells that express HAND1 or BMP4 transcripts (Fig 8D and 8E). They are thus mesoderm, as hypothesized in sheep [14]. At this stage however (Fig 8E), an antero-posterior axis may be accounted for by a thinner posterior pole. Unfortunately, no antibody against the bovine Brachyury protein was available to allow us to test whether a streak was being formed simultaneously on the same ED (instead of on another embryonic disc of a similar stage). Unlike in pig and rabbit embryos, the ED was not positive for *BMP4* (although it was at an early D13-14 stage; unpublished data) and the ring of *BMP4* ^*+*^ cells was not prominent in any direction. Whether this observation may indicate the presence of a new structure for anterior pre-gastrulation differentiation [61] awaits further studies.

## Conclusion

Taken together, this work integrates *in vitro*, *in vivo*, and *in silico* data; bridges the fields of cell biology and EE development; and proposes new gene cores that will open avenues to distinguish EE subtypes or enable functional studies of isolated EE cell types. All of these are necessary steps for future refined characterizations [62] and cell fate studies, in which ECM geometries also take part in cellular differentiation [63].

In this respect, our results on early EE mesoderm cells shed light on an *in embryo* phenomenon in which EE mesoderm expands in a radial mode, but in a slightly different way from that observed in rabbits or pigs, and thus pave the way for future studies on the anterior differentiation of the embryonic tissues at the onset of gastrulation, maybe as early as D12- 13. Coming out at a time when mesoderm precursors appear to be the earliest committed cells to exit pluripotency [63], some of our mesoderm core markers may help in evaluating EE mesoderm involvement in bovine stem cell differentiation studies (iPS [64]).

In this work, we also started deciphering the complexity of EETs pre- and early post-implantation, a period of great importance to studies of livestock and medicine since peri-implantation defects often result in feto-placental pathologies (e.g., IUGR) or miscarriage. Despite this, early EE phenotypes have been poorly described, due to the difficulty of accessing EETs prior to placenta formation and the overwhelming historical focus on the trophoblast, which forms the major uterine interface and placental component.

Because of the ease of access to EETs in the bovine model, we are able to show that the complexity of these tissues originates in their cell types (specific proliferative and adhesive properties, unique molecular signatures) and hypothesize, in view of conserved epithelial-mesenchyme interactions in other tissues ([59], Fig 8), that it may also derive from the interactions of the cell types. If so, bovine and human models may instruct each other much more than expected at first glance.

## Materials and Methods

Embryo collection, cell isolation, western blotting, microarray analysis, RT-PCR, immunostaining and imaging, whole-mount *in situ* hybridization in S3 and S4 Files.

### Real-time impedance curve

Real-time impedance curves of the isolated cells were recorded using the xCELLigence System (Roche). A 96-well E-Plate (Roche) was coated with seven different substrates (all 100 μg/ml): collagen I (Invitrogen; in acetic acid), collagen IV (Invitrogen; in DMEM), fibronectin (Invitrogen; in DMEM), laminin (Invitrogen; in DMEM), Matrigel (BD Biosciences; in DMEM; at this concentration, Matrigel does not form a gel), poly-L-lysine (Sigma; in water), and vitronectin (Invitrogen; in water). Substrates were left for 2 hr at room temperature, and then rinsed with DMEM, left to dry overnight at 4°C, and rinsed again in DMEM before plating of the cells. The cells (50,000/well for trophoblast and 5,000/well for endoderm/mesoderm) were seeded in triplicates onto the 96-well E-Plate. Impedance was measured every 5 minutes during the first 16 hours and then every 30 min for 3 days. Background measurement was performed on 100 μl DMEM medium that contained 10% fetal calf serum (FCS).

### Micro-dissection of the three *in vivo* extra-embryonic cell populations

To isolate the three extra-embryonic cell populations, frozen D18 bovine conceptuses were sectioned into 10-μm slices. From these, the trophoblast, extra-embryonic endoderm, and extra-embryonic mesoderm cells were micro-dissected, using the laser pressure catapulting technique adapted from a previous study [65]. Briefly, 4–6 serial frozen sections of D18 bovine conceptuses were mounted onto ready-to-use PALM membrane slides [0.17-mm polyethylene naphthalate (PEN)] and stored on ice until microdissection. The Robot- MicroBeam (PALM) focused the laser (60 nm) on the specimen, with appropriate energy settings to enable the catapulting of the selected areas into the microfuge cap. Samples were covered with 100 μl of TRIzol (Invitrogen) and stored at -80°C. For RNA extraction, 100 μl of TRIzol and 40 μl of chloroform were added to each sample, mixed, and centrifuged for 15 min at full speed at 4°C. The supernatant was measured and transferred in a new tube, and then the same volume of 70% ethanol was added. Samples were mixed by pipetting. Purification of total RNA was performed with the RNeasy Mini Kit (Qiagen) according to the manufacturer’s protocol.

### Flow cytometry

The concentration of cells in suspensions (after plating for bTC or directly after isolation on the Percoll gradient for bXMC) was determined and 1x10^6^ cells were transferred to fluorescence-activated cell sorting (FACS) polypropylene tubes, incubated with labeled primary antibodies, and analyzed on a FACS LSRII flow cytometer (BD Biosciences). Color compensation was preliminarily set by using Compbeads (BD Biosciences). Two-color live-gating acquisition was used to optimize settings and acquire data. Up to 10,000 events were collected and stored electronically for subsequent analysis with DIVA and FlowJo software. A control tube for each of the chromogens used contained equivalent amounts of isotype standards.

## Supporting Information

S1 Fig Experimental design and key pieces of data. To begin deciphering extra-embryonic complexity prior to placenta formation, we isolated bovine extra-embryonic subtypes at Day 18 (D18), three days prior to implantation (D21), and characterized them using *in vivo*, *in vitro*, and *in silico* methods. The abbreviations used in the figure but not defined within the text are as follows: IF—immuno-fluorescence; MNC—mono-nucleated cells; BNC–bi-nucleated cells.
(TIF)

S2 Fig *In vitro* and *in vivo* extra-embryonic endoderm phenotypes at D18. F-actin and DAPI labeling of endoderm cells (A) *in vitro* and (B) *in vivo*. In (B): large-field images captured through MosaiX acquisition (upper panel) and pan-keratin labeling (lower panel). (C) The evolution of endoderm cell area *in vivo*, computed in stages [from D7-D9 (spherical blastocysts) to D18 embryos (filamentous conceptuses)] from measurements of endoderm cell areas and counts of trophoblast cell numbers from MosaiX images. Similar characteristics have been reported from mono- and multi-nucleated cell types in sheep [71]. Included are the typical elongating stages described in [27, 74]: ovoid (D12-D13), tubular (D14), and early filamentous (D15-D18).
(TIF)

S3 Fig Motility movie, bXMCs, World Cell Race 2011. (MP4)

S4 Fig Flow cytometry analysis of CD56+ cells, reported as the earliest human mesoderm precursors *in vitro* [33] and representing 10% of *in vivo* D18 EET. See also Fig 7B.
(TIF)

S1 File Gene lists corresponding to the D18 micro-dissected EE cell types (Fig 1C). 186 common genes between the three cell types-trophoblast, ExEndoderm and ExMesoderm- (Table 1). 262 common genes between Trophoblast and ExMesoderm (Table 2). 401 common genes between ExEndoderm and ExMesoderm (Table 3). 1193 common genes between Trophoblast and ExEndoderm (Table 4). 596 genes only expressed by ExEndoderm (Table 5). 867 genes only expressed by ExMesoderm (Table 6). 2545 genes only expressed by Trophoblast (Table 7).
(XLS)

S2 File Gene IDs corresponding to DEG cores. For each list, all Expressed Sequenced Tags (ESTs) have been listed (Genbank accession number, GB), as not all have a gene ID (HUGO term). Core Trophoblast (Table 1). Core Endoderm (Table 2). Core Mesoderm (Table 3). Core Epithelium (Table 4).
(XLS)

S3 File Primers (Table 1) and Antibodies (Table 2). (XLS)

S4 File Methods detailed elsewhere [75–82]. (DOC)
