# Supplementary figures and images for: Primary Bovine Extra-Embryonic Cultured Cells: A New Resource for the Study of In Vivo Peri-Implanting Phenotypes and Mesoderm Formation
Source: PLoS One. 2015 Jun 12;10(6):e0127330. doi: 10.1371/journal.pone.0127330 (PMC4466545; doi:10.1371/journal.pone.0127330)

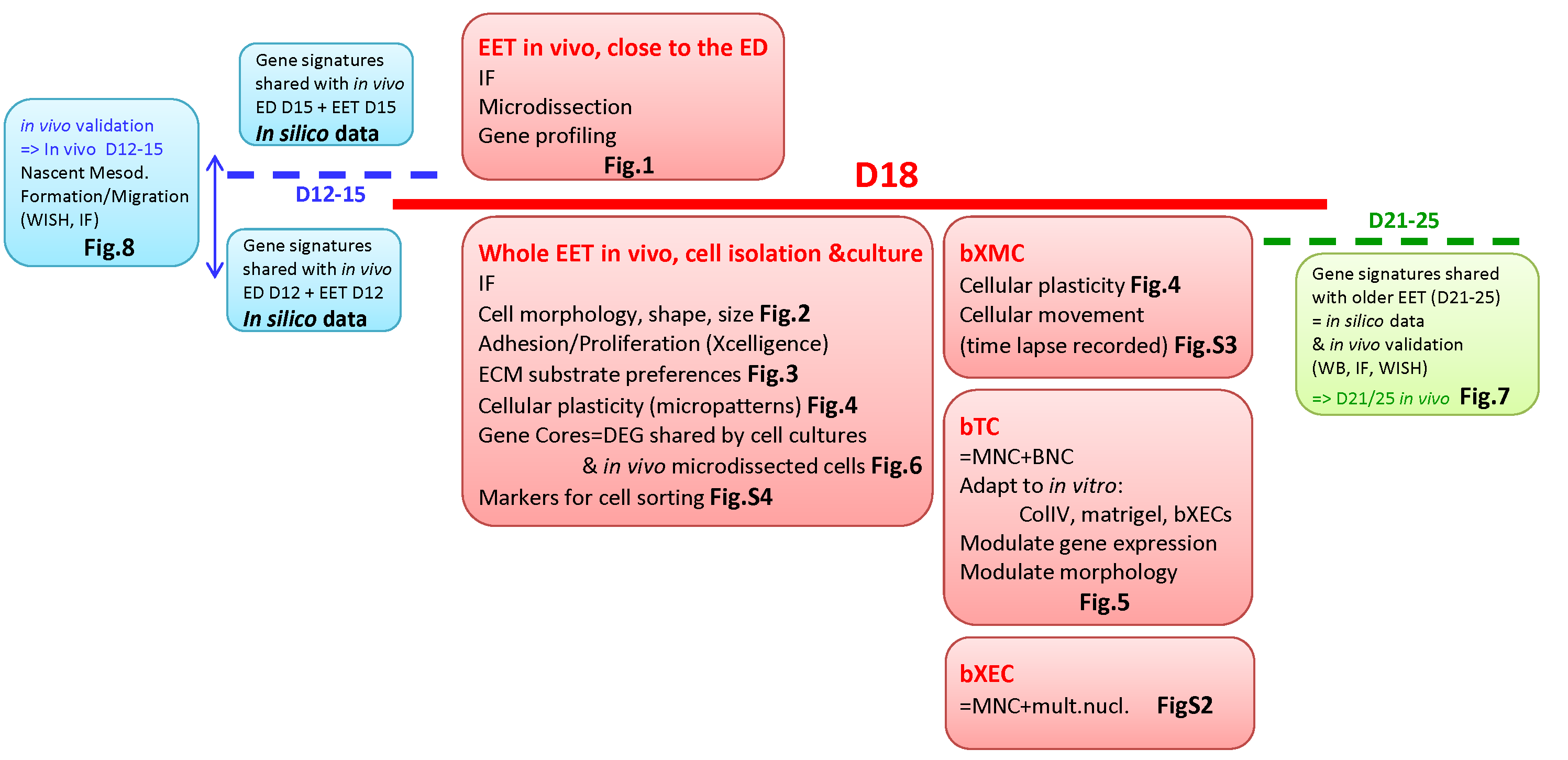

Supplement: S1 Fig — To begin deciphering extra-embryonic complexity prior to placenta formation, we isolated bovine extra-embryonic subtypes at Day 18 (D18), three days prior to implantation (D21), and characterized them using in vivo, in vitro, and in silico methods. The abbreviations used in the figure but not defined within the text are as follows: IF—immuno-fluorescence; MNC—mono-nucleated cells; BNC–bi-nucleated cells. (TIF) [file pone.0127330.s001.tif]

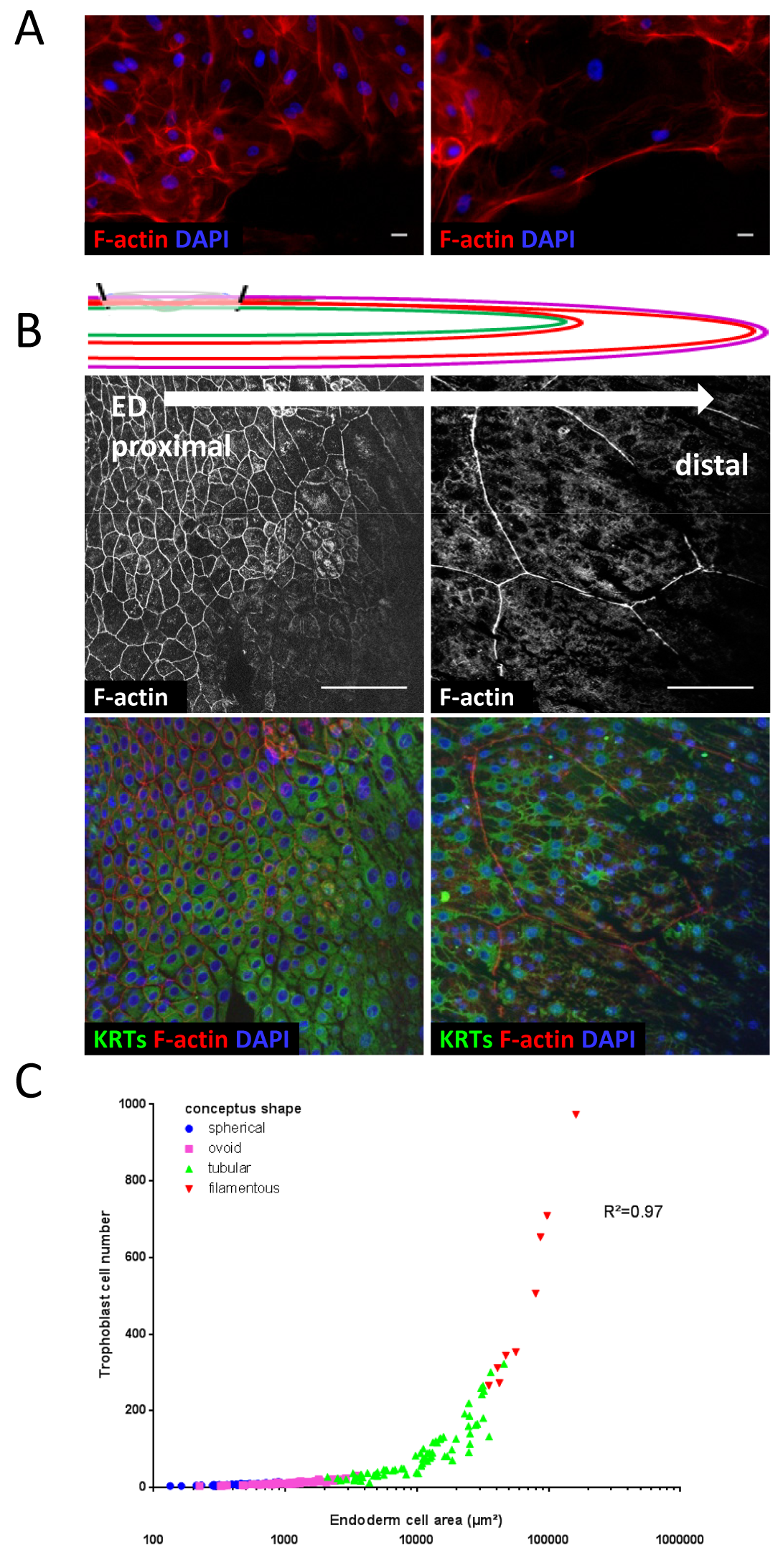

Supplement: S2 Fig — F-actin and DAPI labeling of endoderm cells (A) in vitro and (B) in vivo. In (B): large-field images captured through MosaiX acquisition (upper panel) and pan-keratin labeling (lower panel). (C) The evolution of endoderm cell area in vivo, computed in stages [from D7-D9 (spherical blastocysts) to D18 embryos (filamentous conceptuses)] from measurements of endoderm cell areas and counts of trophoblast cell numbers from MosaiX images. Similar characteristics have been reported from mono- and multi-nucleated cell types in sheep [71]. Included are the typical elongating stages described in [27, 74]: ovoid (D12-D13), tubular (D14), and early filamentous (D15-D18). (TIF) [file pone.0127330.s002.tif]

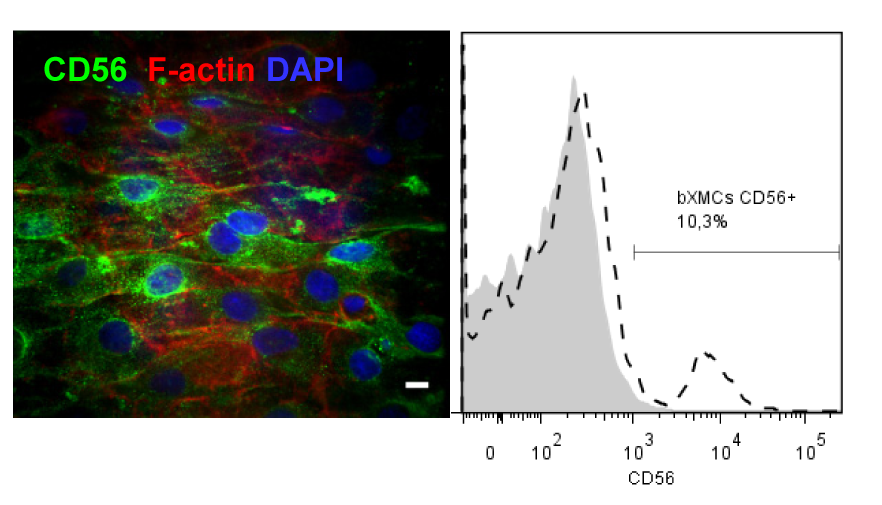

Supplement: S4 Fig — See also Fig 7B. (TIF) [file pone.0127330.s004.tif]
