# Supplementary material for: Primary Bovine Extra-Embryonic Cultured Cells: A New Resource for the Study of In Vivo Peri-Implanting Phenotypes and Mesoderm Formation
Source: PLoS One. 2015 Jun 12;10(6):e0127330. doi: 10.1371/journal.pone.0127330 (PMC4466545; doi:10.1371/journal.pone.0127330)
Supplement: S4 File — (DOC) [file pone.0127330.s008.doc]

**Embryo collection**

Bovine conceptuses were mainly collected at Day 18 post-insemination (or dpi; day of insemination as day 0; n=160), with a few extra samples at 12 dpi (n=4), 15 dpi (n=13), and 21 dpi (n=32) [12]. Samples at 25 dpi (n=9) were collected at the slaughterhouse, by uterine flushing. For each D18 conceptus, the embryonic disc was dissected out and the extra-embryonic tissues stored in DMEM supplemented with 10% fetal calf serum (FCS), on ice, until tissue digestion. Animal use and care were performed in accordance with the International Guiding Principles for Biomedical Research involving Animals at the INRA experimental farm (registered under N° FRTB910 in the national registry) and the protocols for these studies were approved by the local Ethics Committee (Comité d’Ethique en Expérimentation Animale du Centre INRA de Jouy-en-Josas et AgroParisTech (or COMETHEA), registered as 12/084 and 12/086 in the National Ethics Committee registry).

**Isolation of bovine extra-embryonic cell types: bTCs, bXECs, and bXMCs**

In total, 160 conceptuses were used to derive EE cell cultures at D18; 15 independent isolation and culture procedures were performed, each starting from 10 to 12 embryos. Each derivation included the three cell types, two to three substrates, and four time points. All primary cultures were grown for up to 1 week. bTCs, bXECs, and bXMCs were isolated as previously described for human trophoblast cells [75]. Briefly, the extra-embryonic tissues were digested with a sterile 0.05% (w/v) collagenase/trypsin solution (Gibco Life Sciences) at 37°C. The digested tissues were centrifuged and the supernatant was discarded. The pelleted cells were resuspended in DMEM and filtered through a sterile 70-µm filter (BD Biosciences) prior to separation on a 55%-20% Percoll density gradient. All fractions were collected and cell viability was determined with Trypan blue dye (Invitrogen). bTCs were grown either on collagen IV- or Matrigel-coated culture dishes [46, 76], whereas bXECs and bXMCs were grown directly on plastic culture dishes [37, 77] in DMEM (supplemented with 10% FCS, 2 mM glutamine, 100 IU/ml penicillin, and 100 µg/ml streptomycin). bTC supernatants were collected, centrifuged at 200xg for 10 min at 4°C and kept frozen at -20°C until used. bTCs, bXECs, and bXMCs were scraped off the culture dishes, put into tubes, and centrifuged. The supernatants were discarded and the cell pellets snap-frozen and stored at -80°C until used.

**Western blot analysis**

**Preparation of conditioned media.** The conditioned media were prepared for the detection of bIFN-τ release from cells as follows. Cells were cultivated in DMEM + 10% FCS on collagen IV- or Matrigel-coated plates (for trophoblast cells) or directly on plastic (for endoderm and mesoderm cells). The collected media were processed as described in [78]. Briefly, the media were centrifuged in an Amicon Ultracel 50K centrifugal filter unit (Millipore) at 4000 rpm for 1h30 at 4°C. The recovered retentate was further centrifuged on an Amicon Ultracel 3K column (Millipore) at 14000g for 20 min at 4°C to a final concentration of approximately 1000-fold.

**Western blotting procedures.** The concentrated protein samples (approximately 1/5 of final volume) were electrophoresed in the presence of DTT with heat denaturation (at 70°C for 10 min) on a 10% Tris-Bis gel (200V for 30 min), and transferred to nitrocellulose membranes (110V for 1h30 at 4°C). After blocking with TBST-5% skim milk for 1h at room temperature, the membrane was incubated with anti-IFN-τ (kindly provided by M. Guillomot, INRA, UMR BDR; diluted 500-fold) overnight at 4°C. The specific band was revealed by chemiluminescence (West Pico Chemiluminescent; Pierce, Rockford, IL) after 1h incubation at room temperature with an anti-rabbit-peroxidase-coupled antibody (Jackson ImmunoResearch, West Grove, PA).

**Microarray analysis**

Total RNA was isolated using the RNeasy Mini Kit with in-column DNase digestion (Qiagen) and T7 linear amplification was performed using the MessageAmp aRNA kit (Ambion) [34] after bias assessment [79]. Array hybridization was also as described in [26, 34]. Brieﬂy, 500 ng of ampliﬁed RNA (aRNA) were labelled with [α33P] dATP by reverse-transcription and hybridized to an INRA bovine 10K array (GPL7417). Arrays were then exposed to phosphor-screens for 7 days. The hybridization signals were quantiﬁed with Imagene 5.5 software (BioDiscovery) on the PICT/ICE platform. These datasets are available in the Gene Expression Omnibus database (http://www.ncbi.nlm.nih.gov/geo, GSE52967). Data were log-transformed and mean-centered. Statistical and clustering analyses were performed using TIGR MeV 4.7.3 (MultiExperiment Viewer program, <http://www.tm4.org/mev.html>). Differences between cell types were assessed by an ANOVA, with an adjusted Bonferroni correction, so that significance was considered to be P<0.01. An unsupervised hierarchical clustering, based on Euclidean distance and complete linkage, was performed on the significant gene expression differences between the three cell types. Only qualitative analyses were performed on D12 and D15 tissues (ED and EET), and thus were not submitted to the GEO database. For D18-D25 EET analyses, see previous work (GSE13013).

**Functional analysis**

Ingenuity Pathway Analysis software (IPA, <http://www.ingenuity.com/>) was used to compute the statistical differences in gene sets between the three extra-embryonic cell types and define the top functions involved.

**Semi-quantitative RT- PCR**

The reverse transcriptions were done using 1 µg of aRNA in a 20-µl volume using 200 U of Superscript II (Invitrogen) and 1 µl of the second strand primer from the MessageAmp aRNA kit (Ambion), as in [80]. Reverse transcription was performed in triplicate and pooled prior to PCR. All PCRs were carried out using Phusion DNA polymerase according to the manufacturer’s instructions (Finnzymes) and conducted on a Piko PCR machine (Finnzymes). Thermal cycling conditions consisted of an initial denaturing step at 98°C for 1 min, followed by cycles of 10 sec at 98°C, 15 sec at the annealing temperatures of the primer pairs (Table 1 in S3 File), and 30 sec at 72°C. The final step consisted of a 5-min extension at 72°C. Amplified products were fractionated by agarose gel electrophoresis and visualized after scanning (FLA3000, Fuji). Each PCR product was sequenced and checked for its gene ID before being deposited to the NCBI database.

**Immunostaining and imaging**

Extra-embryonic cells or tissues were stained as described in [25]. Briefly, cells/tissues were permeabilized with 0.5% Triton X-100 for 10 min, and then blocked for 1h with 7% donkey serum. Cells/tissues were incubated overnight with the primary antibody, then washed three times in 1X PBS/ 0.1% Tween (each 5 min), and incubated for 1h with secondary antibodies. After three washes in 1X PBS/0.1% Tween (each 5 min), cells/tissues were counterstained and covered with Vectashield that contained DAPI (Vector Labs, Burlingame, CA). Observations were performed using an Observer.Z1 microscope (Zeiss) with a Plan-Apochromat 40x/1.4 oil objective with Apotome module and Axiovision Software (version 4.6). As a negative control, cells/tissues were incubated with the secondary antibodies alone. The antibodies are listed in Table 2 in S3 File.

**WISH**

The isolated embryonic discs were fixed in 4% paraformaldehyde and hybridized to DIG-labeled riboprobes as described in [81]. BMP4, BRACHYUYRY, and HAND1 cDNA fragments originate from earlier work [26, 27, 82].
